# A Key Mediator and Imaging Target in Alzheimer’s Disease: Unlocking the Role of Reactive Astrogliosis Through MAOB

**DOI:** 10.1007/s13139-023-00837-y

**Published:** 2024-01-15

**Authors:** Min-Ho Nam, Heesu Na, C. Justin Lee, Mijin Yun

**Affiliations:** 1 Brain Science Institute, Korea Institute of Science and Technology, Seoul, Republic of Korea; 2 Center for Cognition and Sociality, Institute for Basic Science, Daejeon, Republic of Korea; 3 Department of Nuclear Medicine, Severance Hospital, Yonsei University College of Medicine, Seoul, Republic of Korea

**Keywords:** Alzheimer’s disease, Reactive astrogliosis, Astrocytes, Monoamine oxidase B, PET tracer

## Abstract

Astrocytes primarily maintain physiological brain homeostasis. However, under various pathological conditions, they can undergo morphological, transcriptomic, and functional transformations, collectively referred to as reactive astrogliosis. Recent studies have accumulated lines of evidence that reactive astrogliosis plays a crucial role in the pathology of Alzheimer’s disease (AD). In particular, monoamine oxidase B, a mitochondrial enzyme mainly expressed in astrocytes, significantly contributes to neuronal dysfunction and neurodegeneration in AD brains. Moreover, it has been reported that reactive astrogliosis precedes other pathological hallmarks such as amyloid-beta plaque deposition and tau tangle formation in AD. Due to the early onset and profound impact of reactive astrocytes on pathology, there have been extensive efforts in the past decade to visualize these cells in the brains of AD patients using positron emission tomography (PET) imaging. In this review, we summarize the recent studies regarding the essential pathological importance of reactive astrocytes in AD and their application as a target for PET imaging.

## Introduction

Astrocytes, a subset of neural cells originating from the ectoderm and neuroepithelium, orchestrate multifaceted homeostasis across the molecular, cellular, network, metabolic, and systemic domains within the healthy brain [1]. In contrast, when subjected to physical or chemical insults, astrocytes undergo a transformation into their reactive state, commonly known as reactive astrocytes, with the purpose of mounting a defense mechanism. While these reactive astrocytes indeed fulfill a defensive role under a mild insult, their capacity to uphold homeostasis becomes compromised under a severe insult [2, 3]. Consequently, the significances of mild and severe reactive astrocytes have garnered substantial attention in recent times, primarily due to their pivotal involvement in the pathogenesis of diverse brain disorders characterized by neuroinflammation and neurodegeneration. This encompassing scope extends to conditions such as Alzheimer’s disease (AD), Parkinson’s disease (PD), white matter stroke, spinal cord injury, and amyotrophic lateral sclerosis [4].

Historically, glial alterations accompanying brain inflammation have been well recognized [5]. Notably, in 1906, Alois Alzheimer, the pathologist credited with the initial characterization of AD, observed and illustrated hypertrophied glial cells alongside fiber tangles and substantial plaque deposits in the brains of AD patients. These observations marked a significant milestone in the understanding of AD pathology. In contemporary transgenic mouse models of AD, distinct glial changes are evident, particularly in regions proximate to amyloid-beta (Aβ) plaques. These changes encompass reactive astrocytes and reactive microglia. While the term “reactive astrocytes” does not denote a uniform astrocytic state, they can be broadly identified by their hypertrophied and more extensively branched morphology, coupled with heightened expression of glial fibrillary acidic protein (GFAP), a prominent constituent of astrocyte intermediate filaments [2]. In addition to GFAP, several other molecular markers have emerged as potential indicators of reactive astrocytes. These include monoamine oxidase B (MAOB), excitatory amino acid transporter (EAAT) 1 and 2, S100B, SRY-box transcription factor 9 (SOX9), signal transducer and activator of transcription 3 (STAT3), complement 3 (C3), lipocalin 2 (Lcn2), and serpin family A member 3 (Serpina3n) [2].

Among the various genetic markers, MAOB has been recently highlighted as a key molecule implicated in mediating astrocyte reactivity and the consequential neuronal damage [6–10]. MAOB, alongside monoamine oxidase A (MAOA), belongs to the class of monoamine oxidase, catalyzing the oxidation of monoamines at the outer mitochondrial membrane. MAOB is widely distributed throughout the brain, encompassing regions such as the cerebral cortex, cerebellum, hippocampus, and midbrain [11, 12]. Extensive investigations have revealed that MAOB is primarily localized within astrocytes across most brain regions, with the exception of specific areas such as the dorsal raphe and tuberomammillary nucleus [13, 14]. Traditionally recognized as a participant in the breakdown of monoamine transmitters, including dopamine, serotonin, and norepinephrine, MAOB’s role in regulating the levels of these transmitters has been more recently attributed to MAOA [14, 15]. On the other hand, MAOB, predominantly expressed in astrocytes, controls the tonic inhibition level by mediating astrocytic γ-aminobutyric acid (GABA) synthesis [15].

More notably, astrocytic MAOB has recently emerged as a key pathological molecule in various brain disorders, including AD [6–9, 16], PD [10, 17, 18], and stroke [19]. In particular, a series of recent reports have comprehensively delineated the pathological role of reactive astrocytes through MAOB in AD. Elevated MAOB levels mediate aberrant GABA synthesis in astrocytes, leading to an excessive suppression of neighboring neuronal activity. Simultaneously, during the process of MAOB-mediated GABA synthesis in astrocytes, H_2_O_2_ is generated as a byproduct, inducing oxidative stress that critically contributes to the degeneration of the neighboring neurons and further exacerbates the astrocytic reactivity. These two major cascades are thought to underlie the MAOB-mediated neurodegeneration and neuronal dysfunction in AD.

Due to the profound impact of reactive astrocytes on brain pathologies, extensive efforts have been made over the past decade to visualize them in the brains of AD patients using positron emission tomography (PET) imaging. Notably, MAOB has emerged as an effective imaging target for visualizing reactive astrocytes. A series of PET studies employing MAOB PET tracers has demonstrated the presence and temporal progression of reactive astrogliosis in the brains of AD patients. Therefore, this review aims to revisit the pivotal pathological role of reactive astrocytes in AD and their utilization as a PET imaging target.

## Reactive Astrocytes as a Key Mediator of AD

### Appearance of Reactive Astrocytes in AD

**Fig. 1:**
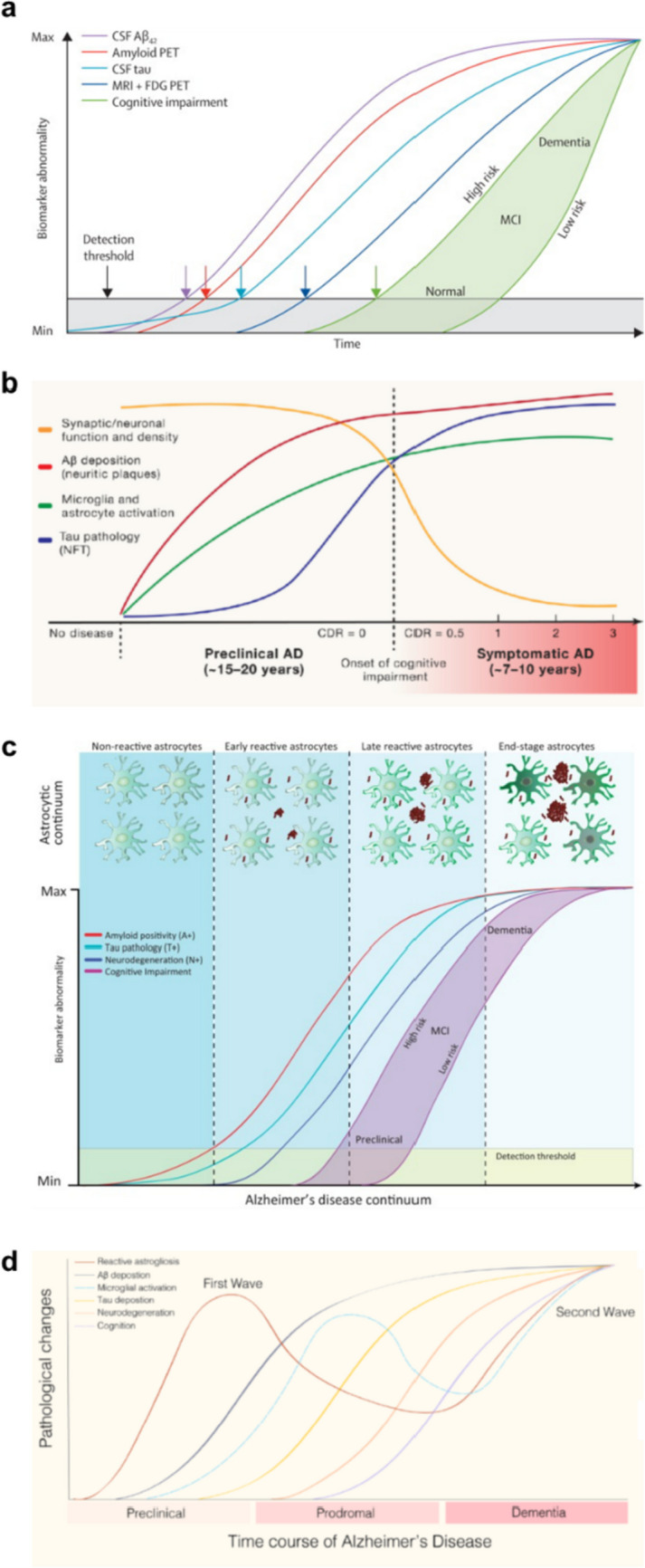
Pathological cascade of AD in relation to clinical course. **a** Schematic diagram about time course of pathological events of AD which omits reactive astrogliosis (original image is from Jack et al., 2013, Lancet Neurology). **b** A schematic diagram depicting astrocytic activation precedes tau pathology but follows Aβ deposition (original image is from Long et al., 2019, Cell). **c** A schematic diagram depicting the progressive astrocyte reactivity during the pathology of AD (original image is from Carter et al., 2019, Trends in Molecular Medicine). **d** A schematic diagram depicting the first and second wave of reactive astrogliosis during the time course of AD (Kumar et al., 2023, Journal of Neurochemistry)

Reactive astrocytes have long been recognized as a byproduct of amyloid beta (Aβ) deposits, tau neurofibrillary tangles, and neurodegeneration in the brains with AD. Historically, these reactive astrocytes were conventionally thought to emerge subsequent to the appearance of other primary pathological hallmarks, relegating their significance in the AD pathology. Accordingly, the reactive astrogliosis was generally omitted when depicting the timing of major AD pathological cascade in relation to clinical course (Fig. 1a) [20].

However, accumulating bodies of recent evidence have unveiled the pivotal role of reactive astrocytes in AD pathology, shedding light on their significant implications. Contrary to prior oversight, the concept of reactive astrogliosis is now being acknowledged as an integral player, prompting a reevaluation of its position in the timeline of major pathological cascade in AD. In 2019, the research group led by David Holtzman suggested that reactive astrogliosis, in conjunction with microgliosis, precedes tau pathology and neuronal dysfunction (Fig. 1b), but follows Aβ deposition [21]. In detail, this review highlighted that activated microglia have the capacity to directly secrete toxic proinflammatory cytokines and indirect mediators that prompt astrocytes to secrete a neurotoxic substance. In essence, this review suggested the harmonious interplay between activated microglia and reactive astrocytes in the pathology of AD, emphasizing that they are not just consequences of neurodegeneration [21]. In the same year, a review article from another group led by Eduardo Zimmer also noted that reactive astrocytes could emerge from the early stage of the pathology [22]. They also illuminated the critical role of reactive astrocytes in the pathology of AD, primarily through their neuroinflammatory actions by releasing cytokines, inflammatory factors, nitric oxide, and reactive oxygen species. Additionally, reactive astrocytes participate in various processes, including the clearance, degradation, or even production of Aβ plaques. While this review does not definitively assert that reactive astrocytes precede other pathological hallmarks such as amyloid and tau pathologies, it appropriately recognizes the antecedent appearance of reactive astrocytes in the progression of neurodegeneration (Fig. 1c) and emphasizes their pathological significance in the context of AD [22].

More recently, in 2021, another research team led by Agneta Nordberg proposed that reactive astrogliosis might manifest at the forefront, even preceding Aβ deposition and microglial activation, as well as tau deposition and neurodegeneration (Fig. 1d), based on observations from PET imaging studies [23]. Of particular significance in this review is the introduction of the concept of the “first (early)” and “second (late)” waves of reactive astrogliosis in AD pathology. Although this notion still needs to be further validated, a series of earlier reports have demonstrated that reactive astrogliosis could be observed through PET imaging prior to positive detection of Aβ or tau PET signals. Certain previous clinical studies even demonstrated a significant elevation in astrocyte reactivity 15–20 years before the onset of clinical symptoms [24, 25]. This evolving perspective underscores the need for comprehensive investigations into the multifaceted contributions of reactive astrocytes within the intricate landscape of AD pathology.

### Pathological Impact of Reactive Astrocytes in AD

Along with the accumulating lines of evidence of earlier manifestation of reactive astrogliosis, the impact of reactive astrocytes on the AD pathology and prognosis has been suggested. A recent report demonstrated that reactive astrocytes can influence Aβ effects on tau pathology in preclinical AD, by assessing serum GFAP level [26]. In detail, individuals with high serum GFAP level showed high correlation between Aβ burden and tau pathology, while individuals with low serum GFAP level showed almost no correlation between the two [26]. In addition, astrocyte reactivity estimated by serum GFAP level was reported to be increased in AD and correlates with cognitive impairment [27]. Furthermore, a more recent report demonstrated that astrocytic reactivity has a power to predict future conversion to AD in patients with mild cognitive impairment [28, 29]. Our group also recently reported that astrocyte reactivity, estimated by acetate PET signal, was significantly correlated with cognitive function in AD patients [7]. Taken together, these recent studies highlight the distinct impact of reactive astrocytes on the prognosis of AD, necessitating the further mechanistic and molecular understanding of reactive astrocyte-mediated pathology.

A previous report proposed a categorization of reactive astrocytes into A1 and A2. Specifically, A1-reactive astrocytes, characterized in the context of lipopolysaccharide (LPS)-mediated neuroinflammation, are suggested to exhibit neurotoxic roles in neurodegenerative disorders such as AD [4]. However, it is important to note that acute neuroinflammation by LPS may not accurately mimic the chronic neurodegenerative conditions. Furthermore, this binary categorization of reactive astrocytes has faced challenges from a recent consensus paper [2]. Instead, the evolving perspective suggests that reactive astrocytes should be viewed as existing along a continuous and graded spectrum of reactivity in response to external harmful stimuli. Therefore, consequently, to comprehend the pathological role of reactive astrocytes in AD, it could be essential to explore the diverse and heterogeneous reactivity of astrocytes within the disease context.

### Molecular Pathology Through Astrocytic MAOB in AD

We have recently published a series of papers highlighting the critical role of MAOB within reactive astrocytes in the context of AD pathology. In 2014, Jo et al. revealed that astrocytes become reactive in the hippocampus of APP/PS1 mouse model of AD and aberrantly synthesize GABA via the enzymatic action of MAOB [8]. Researchers observed heightened MAOB expression in tandem with increased levels of putrescine, the source of astrocytic GABA [8]. The GABA is released tonically from astrocytes through the Bestrophin-1 channel, triggered by an intracellular Ca^2+^ rise [8]. Consequently, neighboring neurons are tonically inhibited by astrocytic GABA, resulting in diminished synaptic transmission and impaired plasticity, evident by reduced spike probability, impaired long-term potentiation [8]. Moreover, the astrocytic GABA-mediated tonic inhibition of hippocampal neurons culminates in memory impairment. These pathological cascades encompassing astrocyte reactivity and synaptic dysfunction were substantially ameliorated with the administration of selegiline, an irreversible MAOB inhibitor [8]. This underscored the significance of astrocytic MAOB in the AD pathology and memory deficits observed in the AD mouse model.

In 2020, Chun et al. discovered that within the process of producing astrocytic GABA, the MAOB enzyme also generates hydrogen peroxide (H_2_O_2_), thereby contributing to neuronal degeneration and the progression of AD pathology [6]. Employing a reactive astrocyte mouse model induced by the astrocyte-specific treatment of diphtheria toxin (referred to as the GiD model; an abbreviation of GFAP-creERT2 crossed with iDTR mouse line), researchers found that pronounced MAOB activity in the severe reactive astrocytes led to excessive production of both GABA and H_2_O_2_ [6]. The increased level of H_2_O_2_ facilitated the expression of inducible nitric oxide synthase (iNOS), subsequently prompting microglial activation [6]. The interplay between reactive astrocytes and microglia resulted in nitrosative and oxidative stress through nitric oxide (NO) and H_2_O_2_, leading to neuroinflammation and neurodegeneration [6]. This model also exhibited decreased spike probability, reduced brain size, and memory deficits [6]. These findings indicate that severe reactive astrocytes can cause neurodegeneration and the related symptoms. Remarkably, the distinct features in this reactive astrocyte model were reversed when treated with either an MAOB inhibitor or an H_2_O_2_ scavenger [6], thus emphasizing the critical role of MAOB-mediated H_2_O_2_ in the neurodegeneration triggered by reactive astrocytes.

Furthermore, these findings gained heightened significance through the recapitulation of the pathological role of MAOB-mediated H_2_O_2_ in the APP/PS1 mouse model of AD [6]. Reactive astrocytes in this animal model generate copious amounts of H_2_O_2_, severely affecting neuronal health. Moreover, the induction of reactive astrocytes using diphtheria toxin was sufficient to provoke tau pathology and substantial neurodegeneration—the pathological hallmarks of human AD—in the APP/PS1 transgenic mice, which do not normally exhibit tau pathology and such severe neurodegeneration. This strongly implies that reactive astrogliosis plays an even more crucial role in human AD pathology. Collectively, these insights underline the central role of reactive astrocytes in AD pathology, mediated by MAOB-induced GABA and H_2_O_2_.

### MAOB as a Therapeutic Target for AD

Based on these findings, MAOB has recently emerged as a potential alternative therapeutic target, diverging from the traditional focus on Aβ and tau. However, several clinically available irreversible MAOB inhibitors, such as selegiline, were previously evaluated in AD patients but yielded unsatisfactory outcomes [30]. A recent study has elucidated the rationale behind the ineffectiveness of selegiline in AD patients [9]. This originates from the fact that irreversible MAOB inhibitors form a permanent covalent bond with flavin adenine dinucleotide (FAD), the cofactor of MAOB, resulting in its permanent destruction. Therefore, long-term administration of selegiline triggers a compensatory mechanism involving diamine oxidase (DAO). Consequently, DAO-mediated astrocytic GABA and H_2_O_2_ synthesis persist, leading to a persistent suppression and degeneration of neighboring neurons in the presence of selegiline.

In response to this challenge, researchers have developed a novel MAOB inhibitor, named KDS2010, which has shown its high therapeutic potential due to its reversibility, selectivity, safety, and potent action [9]. Notably, this inhibitor has demonstrated remarkable therapeutic efficacy in memory recovery of AD model mice, marking a significant step forward in the quest for effective AD treatments [9].

### A Pathological Factor Triggering Astrocytic MAOB in AD: Astrocytic Urea Cycle

Based on previous findings that demonstrate the elevation of MAOB activity within reactive astrocytes as the key pathological mediator of AD, understanding the underlying mechanism of how MAOB activity increases within the context of AD pathology has become crucial. A recent study has presented evidence for the existence of the urea cycle in reactive astrocyte for the first time, revealing that the facilitation of the urea cycle induces reactive astrogliosis and elevates astrocytic MAOB levels in the brains of individuals with AD. Researchers discovered that astrocytes take up Aβ for autophagic degradation, and during this process, excessive aspartate is produced. Subsequently, the excessive aspartate, together with Aβ-derived ammonia boosts the astrocytic urea cycle, which can convert toxic ammonia into less toxic urea. The levels of genes encoding key enzymes constituting the urea cycle, such as arginase 1 (ARG1; encoded by *Arg1*) and ornithine decarboxylase 1 (ODC1; encoded by *Odc1*), are indeed increased in AD. The consequence of boosting the urea cycle is the accumulation of ornithine, which is then enzymatically converted to putrescine by ODC1. The increased level of putrescine finally causes MAOB-mediated GABA and H_2_O_2_ synthesis. These findings were confirmed by the observation that astrocyte-specific gene-silencing of *Odc1* and *Arg1* blocked astrocytic MAOB-mediated AD pathology in the APP/PS1 transgenic mice.

Unlike ARG, which is critically involved in the detoxification of ammonia through the urea cycle, ODC1 acts as a bridge between the urea cycle and detrimental putrescine degradation pathway which produces GABA and H_2_O_2_. Therefore, inhibiting ODC1 does not disrupt the functional integrity of the urea cycle, which is responsible for ammonia removal. Instead, it selectively targets the harmful putrescine degradation pathway, thereby preventing the formation of reactive astrocytes by halting MAOB-mediated production of GABA. Furthermore, Odc1 gene-silencing resulted in a significant decrease in the number of Aβ plaques. This observation suggests that ODC1 could represent a therapeutic target for AD by curbing Aβ-mediated astrocyte reactivity while concurrently maintaining ammonia detoxification. Taken together, these results unveiled the boosted astrocytic urea cycle as the key pathological factor that triggers astrocytic MAOB elevation in AD.

### Metabolic Supports of Reactive Astrocyte-Mediated AD Pathology Through Acetate

Given the pivotal role of astrocyte reactivity in AD pathology, it becomes imperative to address the question of how these reactive astrocytes are sustained metabolically. A recent study has shed light on this matter, reporting that reactive astrocytes exhibit heightened uptake of acetate via monocarboxylate transporter 1 (MCT1), the expression of which is augmented during the induction of astrocyte reactivity [7]. Acetate, in turn, promotes the urea cycle and MAOB-mediated GABA synthesis in astrocytes, although the precise underlying mechanism remains elusive. Notably, the enhanced astrocytic GABA due to acetate intake exerts a suppressive influence on regional glucose metabolism and the activity of neighboring neurons [7].

The study further demonstrated that astrocyte-specific gene-silencing of MCT1 reversed the cascade triggered by astrocytic MAOB in the AD mouse models. This encompassed the attenuation of reactive astrogliosis, the mitigation of neuronal activity suppression, and the amelioration of spatial memory deficits [7]. These findings strongly imply that acetate serves as a metabolic support for the pathological processes of reactive astrocytes via MAOB in the AD brain. Further investigations will be necessary to comprehensively understand the intricate metabolic interplay between reactive astrocytes and degenerating neurons.

## Reactive Astrocytes as an Imaging Target of AD

One promising way for enhancing research in reactive astrocyte is the use of PET radiotracers, such as ^11^C-deutrium-l-deprenyl (^11^C-DED), ^11^C-BU99008, ^18^F-THK5351, ^11^C-acetate, and ^18^F-SMBT-1 [23]. These imaging biomarkers have the potential to provide valuable insights into astrocyte reactivity in living brains as well as postmortem brain tissues. Despite the non-specificity to astrocytes, MAO-B has been a major target to visualize reactive astrocytes. Of the radiotracers targeting MAO-B, ^11^C-DED has been most used to find the cross-link between Aβ, tau, and reactive astrogliosis [31–33]. In postmortem studies, there has been a positive correlation between astrogliosis and Aβ deposition in AD related brain regions. In addition, postmortem autoradiography studies with ^3^H-THK5117 (a PET radiotracer for tau accumulation) and ^3^H-DED showed a close correlation between astrogliosis and tau aggregation [32]. However, there has been a concern whether THK5117 is a radiotracer for tau aggregates and further studies would be needed to elucidate the association between astrogliosis and tauopathy with well-validated radiotracers. Nevertheless, there has been a previous study reporting the role of severe astrogliosis leading to tauopathy in neurons [6].

^11^C-DED PET studies in presymptomatic AD carriers and micro-PET studies in APP Swedish mutation (APPswe) transgenic mice models have showed increased reactive astrogliosis at earlier stages of AD progression preceding the early Aβ deposition in brain. In patients, the reactive astrogliosis was significantly elevated even 15–20 years before the onset of clinical symptoms followed by a decline with increasing Aβ accumulation and disease progression [24, 25]. Furthermore, there was a negative correlation between ^11^C-DED binding and cerebral glucose metabolism on ^18^F-fluorodeoxyglucose (^18^F-FDG) PET [34]. In addition, there were dynamic changes in DED binding in which there was an increased DED binding at prodromal stages, lower DED binding at AD dementia and then, at advanced stage of AD dementia (postmortem), increase again in DED binding [23]. The cellular and molecular mechanisms for the findings need further investigation in the future. Other than ^11^C-DED, there are studies with a new MAO-B PET radiotracer, specifically SMBT-1 which may shed light on understanding the impact of reactive astrocytes in AD pathogenesis [35, 36].

BU99008 is a novel tracer which targets the mitochondrial imidazoline_2_ binding sites (I_2_Bs) predominantly expressed in the outer mitochondrial membrane of the astrocytes and to a lower extent in neurons [37]. I_2_Bs have been related to GFAP regulation [38] and have shown to be co-expressed with MAO-B in the human frontal cortex during the process of aging [39]. ^3^H-BU99008 could visualize reactive astrogliosis in AD postmortem brains with good specificity and selectivity [40]. Interestingly, frozen large brain section autoradiography showed differences in the binding characteristics of ^3^H-BU99008 and ^3^H-DED, which may represent astrocyte subtype heterogeneity in AD progression [40] or an off-target binding of ^3^H-BU99008.

As mentioned, acetate is taken up via MCT1 and serves as a metabolic support for the pathological processes of reactive astrocytes via MAOB. There have been recent reports on the use of ^11^C-acetate PET in detecting reactive astrocytes in patients with multiple sclerosis [41]. Recently, Nam et al. reported the potential value of PET imaging with ^11^C-acetate and ^18^F-FDG by visualizing reactive astrogliosis and the associated neuronal glucose hypometabolism in the brains with neuroinflammation and AD [7]. The elevated acetate uptake in reactive astrocytes boosts the aberrant astrocytic GABA synthesis when amyloid-beta is present. The excessive astrocytic GABA subsequently suppresses neuronal activity and glucose uptake through decreased glucose transporter-3 in the diseased neurons. ^11^C-acetate uptake was significantly increased in the entorhinal cortex, hippocampus, and temporo-parietal neocortex of the AD patients while ^18^F-FDG uptake was significantly reduced in the same regions. Additionally, there was a strong correlation between the patients’ cognitive function and the PET signals of both ^11^C-acetate and ^18^F-FDG.

## Concluding Remarks

Reactive astrocyte plays a crucial role in the pathogenesis of AD at an early stage and throughout the course of disease progression. It is likely to precede other pathological hallmarks, such as amyloid-beta plaque deposition, and significantly affect the entire process of AD-related neuropathological changes, including neuronal glucose hypometabolism, tau aggregation, microglial activation, and neurodegeneration through the production of GABA and H_2_O_2_. Further understanding of astrocyte reactivity will contribute to advances in the comprehension of the disease’s mechanisms and potential therapeutic targets. Given this importance, biomarkers have been developed in the past decade to measure or visualize reactive astrocytes. While PET imaging biomarkers have revolutionized the current understanding of AD by enabling early detection, staging, treatment monitoring, and prediction of clinical outcomes, they can also enhance the accuracy of fluid biomarkers, such as cerebrospinal fluid markers or blood-based assays. Early detection of AD at the prodromal stage using a combination of fluid and imaging biomarkers related to reactive astrogliosis would contribute to early intervention, improved quality of life, cost reduction related to AD patient management, and advancements in research and treatment.

## Data Availability

Data sharing is not applicable to this article as no datasets were generated or analyzed during the current study.
